# Convergent *Klebsiella pneumoniae* strains belonging to a sequence type 307 outbreak clone combine cefiderocol and carbapenem resistance with hypervirulence

**DOI:** 10.1080/22221751.2023.2271096

**Published:** 2023-10-16

**Authors:** Katharina Schaufler, Thaddäus Echelmeyer, Michael Schwabe, Sebastian Guenther, Jürgen A. Bohnert, Karsten Becker, Helmut Fickenscher, Aike Bueter, Gregor Maschkowitz, Andi Krumbholz, Dennis Nurjadi, Stefan E. Heiden, Elias Eger

**Affiliations:** a Institute of Infection Medicine, Christian-Albrecht University Kiel and University Medical Center Schleswig-Holstein, Kiel, Germany; b Institute of Pharmacy, Pharmaceutical Microbiology, University of Greifswald, Greifswald, Germany; c Department of Epidemiology and Ecology of Antimicrobial Resistance, Helmholtz Institute for One Health, Helmholtz Centre for Infection Research HZI, Greifswald, Germany; d University Medicine Greifswald, Greifswald, Germany; e Institute of Pharmacy, Pharmaceutical Biology, University of Greifswald, Greifswald, Germany; f Friedrich Loeffler-Institute of Medical Microbiology, University Medicine Greifswald, Greifswald, Germany; g Labor Dr. Krause und Kollegen MVZ GmbH, Kiel, Germany; h Department of Infectious Diseases and Microbiology, University of Lübeck and University Medical Center Schleswig-Holstein, Lübeck, Germany

## Letter

*Klebsiella pneumoniae* (*K. pneumoniae*) is associated with a variety of severe diseases, including pneumonia and bacteremia [1]. As the strict boundaries between hypervirulent (hvKp) and classical (cKp) pathotypes blur, an increasing number of studies report the spread of difficult-to-treat convergent *K. pneumoniae* [2]. This is mostly driven by hybrid plasmids harbouring both antimicrobial resistance (AMR) determinants, such as genes for carbapenemases (e.g. *bla*_NDM-1_ and *bla*_OXA-48_), and hypervirulence, such as siderophore secretion and internalization (e.g. for aerobactin and its cognate receptor) and hypermucoviscosity. Traditionally, the hvKp pathotype has been identified through a positive string test indicating hypermucoviscosity [3]. This virulence trait confers protection against the host’s immune system and is nowadays more precisely defined by the identification of multiple genetic biomarkers and *in vivo* virulence [4].

The emergence of multidrug-resistant (MDR) *K. pneumoniae* in general and convergent strains in particular calls for alternative therapeutic approaches. Cefiderocol, a cephalosporin with structural homologies to ceftazidime and cefepime and a catecholate siderophore moiety that is taken up by siderophore receptors, seemed very promising upon its medical approval in the United States of America in 2019 and the European Union in 2020 [5]. However, since then, multiple studies have identified different mechanisms conferring resistance to cefiderocol, including the production of carbapenemases such as NDM-1, mutations in porins and siderophore receptor genes (e.g. *ompK36* or *cirA*), and efflux pumps [6]. In general, cefiderocol resistance is seemingly due to a complex interplay of factors, such as the co-production of distinct β-lactamases in combination with decreased membrane permeability or increased drug export [7]. While resistance to last-resort drugs such as carbapenems and colistin in hypervirulent *K. pneumoniae* strains has gained increasing attention in the last years, our understanding of convergent representatives resistant to cefiderocol remains limited.

**Figure 1.**
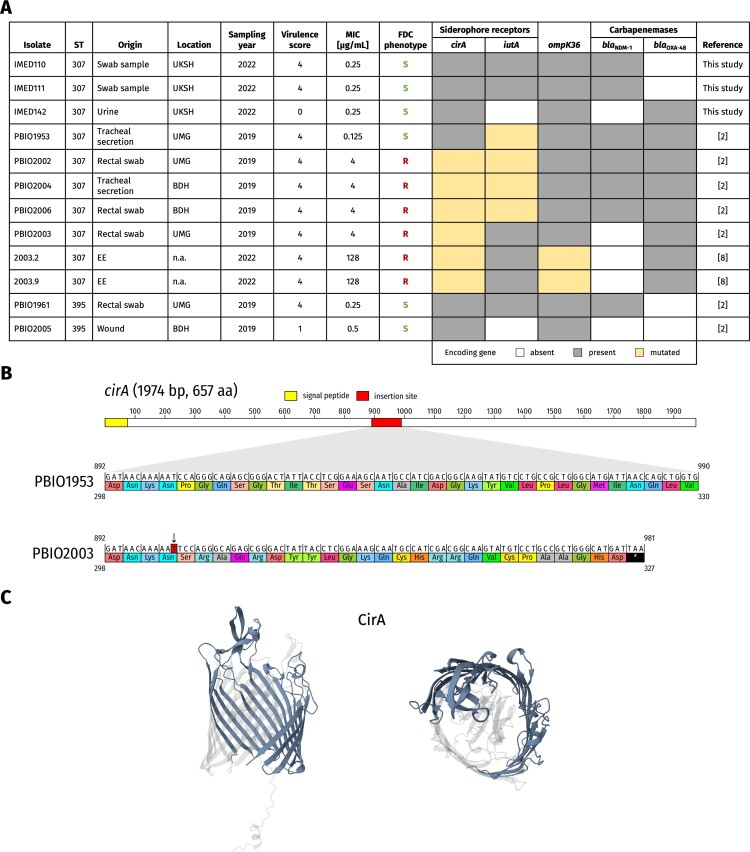
**Overview of investigated strains and their geno- and phenotypic characteristics. A** Metadata, genotypic information, and phenotypic resistance traits of the investigated strains. Minimum inhibitory concentrations for cefiderocol were determined by broth microdilution and interpreted according to EUCAST guidelines. The virulence score was determined using Kleborate, with 0 = negative for yersiniabactin, colibactin and aerobactin, 1 = only yersiniabactin, 2 = only yersiniabactin and colibactin (or colibactin only), 3 = aerobactin (without yersiniabactin or colibactin), and 4 = aerobactin and yersiniabactin (without colibactin). Predictions for siderophore receptors and *ompK36* (highlighted in gray) are based on BLAST using PBIO1953 as reference, whereas predictions for carbapenemase genes are based on alignments of sequences from the AMRFinderPlus database [10] (default settings of identity ≥90.0% and coverage ≥50.0%). Mutations in the gene sequence are highlighted in yellow and uncoloured boxes indicate the absence of the respective gene. **B** Schematic presentation of genetic changes (red) in the *cirA* gene of cefiderocol-resistant PBIO2003. The single thymine base duplication (arrow) results in a frameshift and a premature stop codon (black). **C** Cartoon representation of modelled protein structure of the catecholate siderophore receptor CirA. Predicted changes in the architecture of the siderophore receptor in lateral view (left) and top view (right) are coloured in transparent gray. BDH, Neurorehabilitation centre in Greifswald; EE, experimental evolution; FDC, cefiderocol; MIC, minimum inhibitory concentration; n.a., not applicable; R, resistant; S, susceptible; UKSH, University Medical Centre Schleswig-Holstein; UMG, University Medicine Greifswald.

Here, we report cefiderocol-resistant, convergent *K. pneumoniae* strains of the global sequence type (ST)307 that were isolated as part of a clinical outbreak in Western Pomerania (Germany) between 2019 and 2020. Our previously published studies from 2019 and 2020 demonstrated the clonality and outbreak character of the ST307 strains as well as geno- and phenotypic MDR and hypervirulence characteristics [2, 8]. Now, we additionally determined minimum inhibitory concentrations (MICs) for cefiderocol for selected representatives. In total, four ST307 outbreak strains (PBIO2002, PBIO2003, PBIO2004, and PBIO2006) showed a MIC of 4 µg/mL, which was evaluated by two different testing methods (i.e. agar diffusion assays using MIC test strips and broth microdilution using iron-depleted Mueller-Hinton broth according to EUCAST guidelines [The European Committee on Antimicrobial Susceptibility Testing. 2023. Breakpoint tables for interpretation of MICs and zone diameters. Version 13.0. http://www.eucast.org. Accessed June 01, 2023.]), each with three biological replicates. Interestingly, other ST307 outbreak strains, such as the index strain PBIO1953, were susceptible to cefiderocol with MIC values below the EUCAST breakpoint of 2 µg/mL (0.125 µg/mL). This discrepancy prompted a detailed investigation of the genetic variation among these strains. In addition to the five ST307 strains from the published outbreak study (PBIO1953, PBIO2002, PBIO2003, PBIO2004, and PBIO2006), we analyzed three unrelated and previously unpublished ST307 *K. pneumoniae* representatives from the University Medical Centre Schleswig-Holstein (IMED110, IMED111, and IMED142). We additionally included two ST395 strains (PBIO1961 and PBIO2005) that were also associated with the outbreak. Two PBIO2003–derived mutants (2003.2 and 2003.9) with different mutations in the porin gene *ompK36* were investigated as well (Figure 1). These two mutants have been originally obtained by selecting for ceftazidime/avibactam-resistant representatives in a previously published experimental evolution (EE) study [9].

Figure 1 demonstrates that mutations in the *cirA* gene encoding for the catecholate siderophore receptor were only found in PBIO2002, PBIO2003, PBIO2004, and PBIO2006 (Figure 1A). The mutated *cirA* gene of these four previously published outbreak strains had a single base pair duplication c.903dupT (p.Pro302SerfsX26), resulting in a frameshift and an early stop codon (Figure 1B,C). These strains showed cefiderocol resistance just above the breakpoint (4 µg/mL). Cefiderocol resistance based on *cirA* alterations such as functional loss due to nucleotide change subsequently leading to a frameshift and early stop codon and other, heterogeneous mutations have already been described in EE studies performed for *K. pneumoniae* [11], and in other *Enterobacterales* species during treatment [12]. However, to our knowledge, single base duplications in the *cirA* gene have not yet been described in this context. Notably, these mutations were present prior to the approval of cefiderocol for clinical use in Germany, as the outbreak strains were collected between June 2019 and February 2020 [2, 8].

Second, mechanisms contributing to reduced membrane permeability have synergistic effects on cefiderocol resistance. Here, mutations in the gene encoding for the OmpK36 channel of 2003.2 and 2003.9 resulted in a 32-fold increase in cefiderocol MIC values compared to the *cirA* mutation alone in PBIO2003 (Figure 1A). In contrast to CirA and OmpK36, changes of the aerobactin siderophore receptor IutA associated with hypervirulence is seemingly not directly involved in cefiderocol resistance. That is, the outbreak strains PBIO1953, PBIO2002, PBIO2004, and PBIO2006 revealed a missense mutation in *iutA* c.421G > T (p.Gly141Cys), but only those carrying the additional mutated *cirA* gene showed phenotypic cefiderocol resistance. Also note that IMED142 and PBIO2005 did not carry *iutA* at all and were susceptible (Figure 1A).

Third, the production of carbapenemases such as NDM-1 alone and in the absence of mutations of genes responsible for membrane permeability and siderophore transporters is apparently not sufficient to confer cefiderocol resistance. For example, ST307 strains IMED110, IMED111, and IMED142 as well as the ST395 strain PBIO1961 were positive for either *bla*_OXA-48_ or *bla*_NDM-1_ but carried the wild-type *cirA* allele (no mutation) and exhibited MIC values of 0.25 µg/mL (Figure 1A). Furthermore, the co-production of OXA-48 and NDM-1 by PBIO1953 did not result in resistance or increased tolerance to cefiderocol, as PBIO1953 had the lowest MIC in this study (0.125 µg/mL), which is in line with previous findings [6, 13]. Interestingly, the ST395 strain PBIO2005 had a MIC value of 0.5 µg/mL despite being negative for carbapenemases and the *iutA* gene. Why this is the case remains to be investigated. Also note that there is evidence that while NDM-1 does not contribute significantly to cefiderocol resistance alone, it appears to facilitate *cirA* mutations [7]. These results highlight that the co-occurrence of multiple mechanisms likely leads to cefiderocol resistance.

Finally, all cefiderocol-resistant strains studied (except for the porin mutants derived from PBIO2003 [2003.2 and 2003.9]) exhibited hypervirulent phenotypes, including increased siderophore secretion, hypermucoviscosity, the ability to survive in the presence of human serum and bile salts, and high mortality rates in an *in vivo* infection model as highlighted in our previous studies [2, 9]. Interestingly, this is seemingly regardless of AMR even though AMR acquisition comes at a fitness and virulence cost for the bacterial host, at least immediately after resistance development [9]. Further studies will need to address whether cefiderocol resistance in other bacterial strains is also not at the expense of reduced bacterial fitness and virulence, or whether it is the result of compensatory mechanisms.

In conclusion, this study highlights the clinical relevance of recently emerging, convergent *K. pneumoniae* strains and the need for reliable treatment options. By analyzing a set of convergent *K. pneumoniae* from different backgrounds and with heterogenous geno- and phenotypic composition, we not only address the complex nature of cefiderocol resistance and the continued need for further investigations, but also that AMR is a result of natural evolution, given the fact that PBIO2003 has been isolated before cefiderocol was widely used in Germany. The final verification of the direct effect of the detected *cirA* mutation on cefiderocol resistance will be prospectively performed.

## Data Availability

The data from this study have been deposited in the European Nucleotide Archive (ENA) at EMBL-EBI under accession number PRJEB63064. Additional data from the outbreak strains (PBIO1953, PBIO1961, PBIO2002, PBIO2003, PBIO2004, PBIO2005, and PBIO2006) and porin mutants derived from PBIO2003 (2003.2 and 2003.9) are available under accession numbers PRJEB37933 and PRJEB48690, respectively.
